# Cocaine

**Published:** 1889-06

**Authors:** 


COCAINE.—A peculiar property has been discovered in Cocaine when applied to the nasal passage, that of weakening the sexual power of the patient. The sensation is described by one man as that of *goneness* in the sexual organs. Not unlike that which immediately succeeds the act of coition.

